# Prospective evaluation of NGS-based liquid biopsy in untreated late stage non-squamous lung carcinoma in a single institution

**DOI:** 10.1186/s12967-020-02259-2

**Published:** 2020-02-17

**Authors:** Simon Heeke, Véronique Hofman, Marius Ilié, Maryline Allegra, Virginie Lespinet, Olivier Bordone, Jonathan Benzaquen, Jacques Boutros, Michel Poudenx, Salomé Lalvée, Virginie Tanga, Carole Salacroup, Christelle Bonnetaud, Charles-Hugo Marquette, Paul Hofman

**Affiliations:** 1 Laboratory of Clinical and Experimental Pathology, Pasteur Hospital, Université Côte d’Azur, 30 Avenue de la Voie Romaine, 06000 Nice, France; 2 Hospital-related Biobank (BB-00033-0025), Pasteur Hospital, Université Côte d’Azur, 30 Avenue de la Voie Romaine, 06000 Nice, France; 3 FHU OncoAge, Pasteur Hospital, Université Côte d’Azur, 30 Avenue de la Voie Romaine, 06000 Nice, France; 4 Pulmonary Department, Pasteur Hospital, Université Côte d’Azur, 30 Avenue de la Voie Romaine, 06000 Nice, France

**Keywords:** Liquid biopsy, Non-small cell lung carcinoma, NGS, Driver genomic alterations, cfDNA

## Abstract

**Background:**

NGS from plasma samples in non-squamous cell lung carcinoma (NSCC) can aid in the detection of actionable genomic alterations. However, the absolute clinical value of NGS in liquid biopsy (LB) made at baseline is currently uncertain. We assessed the impact of plasma-based NGS using an in-house test and an outsourced test in comparison to a routine molecular pathology workflow.

**Methods:**

Twenty-four advanced/metastatic treatment-naïve NSCC patients were prospectively included. NGS analyses were conducted both in-house using the Oncomine cfTNA Panel and in an external testing center using the Foundation Liquid assay. NGS analysis and/or specific molecular based assays were conducted in parallel on tissue or cytological samples.

**Results:**

Both LB tests were well correlated. Tissue NGS results were obtained in 67% of patients and demonstrated good correlation with LB assays. Activating EGFR mutations were detected using LB tests in three patients. PD-L1 expression assessed in tissue sections enabled the initiation of pembrolizumab treatment in five patients.

**Conclusion:**

NGS from LB is feasible in routine clinical practice using an in-house or an outsourced test at baseline. However, the impact on therapy selection was limited in this small series of patients and LB was not able to replace tissue-based testing in our hands.

## Background

The detection of genomic alterations in advanced or metastatic stages of non-squamous cell lung carcinoma (NSCC) is crucial in order to select the appropriate treatment strategy [1]. According to the international guidelines, the current mandatory gene panel to evaluate at baseline in NSCC is limited to very few genes including *EGFR*, *ALK*, *ROS1* and *BRAF*, and, in exceptional cases, to include some additional targets (*MET*, *RET*, *NTRK*), even if these latter genes have not been systematically analyzed in treated-naïve patients until now [1]. However, the rapid development of new therapeutic approaches and the urgent need to increase the number of patients in clinical trials requires the testing of additional genomic alterations although not necessarily in routine clinical care nor in the first-line setting.

In patients with tissue material that is insufficient in size or quality to carry out all the planned analyses, liquid biopsies (LB) from plasma samples present an interesting alternative for the analysis of genomic alterations, even if a tissue re-biopsy should be mandatory if possible [2]. Initially, plasma-based testing has been mainly established for the analysis of *EGFR* mutations with good reproducibility but NGS has also recently been developed to allow the analysis of several genes in parallel, with resolution down to 0.1% allele frequency [3, 4]. Today, plasma-based NGS can either be carried out in-house using targeted sequencing panels or blood can be sent out to certified testing centers who provide a final report outlining the detected genomic alterations. An in-house NGS-based LB assay would be advantageous to reduce turn-around time (TAT) and costs. Additionally, having access to raw data is important for data management and bioinformatics analyses in an academic institution. However, the outsourced testing requires no technical equipment and is independent of sample throughput.

Additionally, it is attractive to consider that combined NGS analyses from both circulating free and tissue DNA obtained in the same patient can lead to optimized detection of an actionable genomic alteration, since this latter could be sometimes absent or non-detectable due to heterogeneity in tissue biopsies [5]. However, the absence of a genomic alteration in one of these tests could be linked to the biology of the tumor but might also be limited by the sensitivity of the respective molecular tests, which is additionally dependent on the sample quality.

The purpose of this study was to evaluate the feasibility of implementing plasma-based NGS in routine clinical care using an outsourced test and an in-house sequencing panel in a prospective cohort of 24 unselected NSCC patients. We have compared the concordance of the two LB tests, and we have included molecular tissue testing when possible to confirm the presence of detected mutations. Finally, we assessed the impact of the sequencing results on the treatment strategy to assess the value of NGS from LB in routine clinical care.

## Methods

### Patient selection

Twenty-four patients have been prospectively and consecutively included between January 2019 and July 2019 in a single hospital center (Pasteur Hospital, Nice, France). Patients were included after confirmation of an advanced or metastatic NSCC. Patients were included at baseline in an unselected population and no exclusion criteria were defined. From each patient, 37 ml of blood was taken: 20 ml in EDTA tubes for in-house analysis and 17 ml using the kit provided with the Foundation Liquid assay and directly sent to the certified testing center (Foundation Medicine; Cambridge, MA, USA).

This study was approved by the local ethics committee (Nice Hospital University) and all patients signed an informed consent. This study was performed in accordance with the declaration of Helsinki.

### In-house mutation analysis

For in-house testing, blood was processed within 2 h of phlebotomy. Samples were analyzed using the Oncomine cfTNA assay (Thermo Fisher Scientific, Waltham, MA, USA) according to manufacturer’s instruction. The panel uses TAC-Seq technology [6] to detect mutations in ~ 168 hotspots of the following genes: *ALK*, *BRAF*, *EGFR*, *ERBB2*, *KRAS*, *MAP2K1*, *MET*, *NRAS*, *PIK3CA*, *ROS1*, and *TP53* (Additional file 1: Figure S1). Additionally, it detects copy number variations in the *MET* gene and selected gene fusions of *ALK*, *ROS1* and *RET* [7]. Briefly, total nucleic acids (TNA including cfDNA and cfRNA) were isolated using the MagMAX™ Cell‑Free Total Nucleic Acid Isolation Kit (Thermo Fisher Scientific, Waltham, MA-US) according to manufacturer’s instructions using 2 ml of plasma per patient. The cfDNA concentration was analyzed using the Qubit dsDNA HS Assay Kit (Thermo Fisher Scientific). Libraries were prepared using the Oncomine Lung Cell‑Free Total Nucleic Acid Research Assay (Thermo Fisher Scientific) and loaded on an Ion 530 sequencing chip utilizing an Ion Chef system (Thermo Fisher Scientific). Sequencing was performed on an Ion S5 sequencer running on Torrent Suite v5.6 (Thermo Fisher Scientific). Data was analyzed using the Ion Reporter v5.12 and the Oncomine TagSeq Lung v2 Liquid Biopsy-w2.2 workflow as provided by the manufacturer. The workflow automatically calculates the detection limit for each position based on the sequencing depth and only mutations with an allele frequency superior to the detection limit were considered.

In parallel, biomarker assessment from tissue samples obtained by small biopsies was conducted according to the workflow already established at the Laboratory of Experimental and Clinical Pathology (LPCE, Nice, France) which is certified according to ISO 15189 [8]. Briefly, one tissue Section (5 µm) was analyzed for *EGFR* evaluation using the Idylla system [9], and 4 tissue Sections (3 µm each) were used for ALK (D5F3, Ventana, Tucson, AZ, USA), ROS1 (D4D6, Cell Signaling, Danvers, MA, USA), BRAF (VE1, Ventana) and PD-L1 (22C3, Dako, Santa Clara, CA, USA) immunohistochemistry for status assessment. NGS analysis from DNA extracted from formalin-fixed and paraffin-embedded (FFPE) tissue was conducted using the AmpliSeq Hotspot V2 Panel (Thermo Fisher Scientific) as described previously [10]. Genes covered by the respective panels are summarized in Additional file 1: Figure S1.

### External testing

The Foundation Liquid test (Foundation Medicine, Cambridge, MA, USA) is an outsourced test that analyzes genetic alterations in 70 genes (the full exonic region is covered for 35 genes, and for a further 35 genes only selected exons are analyzed) as well as selected rearrangements in 7 genes (Additional file 1: Figure S1). Additionally, the microsatellite instability status can be analyzed [11]. For the analysis, phlebotomy has to be performed using provided blood tubes. After phlebotomy, samples were sent directly to the certified testing center in Cambridge (MA, USA). The results are represented in a report highlighting the detected mutations including the allele frequency for some loci, and information of clinical trials associated with the detected mutations. No access to raw sequencing data is provided.

### Data analysis

Data was analyzed using R v3.6.1 (R Foundation, Vienna, AT) [12]. Allele frequency correlation was assessed using the coefficient of determination (R^2^). Different turn-around times were calculated using the student’s t test. The study was not defined to show superiority of one diagnostic test over the other and all data shown are descriptive.

## Results

### Implementation of NGS from plasma at baseline in routine clinical care for metastatic or advanced NSCC

In total, 24 samples were tested in-house using the Oncomine cfTNA (cell-free total nucleic acids including both RNA and DNA) panel and 24 were sent out for testing using the Foundation Liquid assay. Patient characteristics are shown in Additional file 1: Table S1. 24/24 (100%) and 23/24 (96%) samples were successfully tested using the in-house and the outsourced assays, respectively. The median concentration of extracted cfDNA was 1.16 ng/µl (range: 0.39–76.60 ng/µl; cfRNA was not analyzed). The median sensitivity for the in-house approach (80% percentile across all amplicons covered) was 0.205% allele frequency (range = 0.102–0.794). Increased cfDNA concentration improved detection limit and approx. 1 ng/µl of cfDNA (approx. 13 ng of cfDNA for the in-house assay in total) was sufficient to reproducibly reach a detection limit of < 0.2% of allele frequency (Additional file 1: Figure S2). Sufficient tissue availability allowed the NGS assessment from tumor sections in 16/24 (67%) patients. The median TAT for the outsourced test was 10 business days (including shipping time; Additional file 1: Figure S3) versus 28 days in the in-house testing which was prolonged as sufficient samples had to be accumulated before a sequencing run (p < 0.001). However, the minimal TAT for the in-house test is 4 business days.

**Fig. 1:**
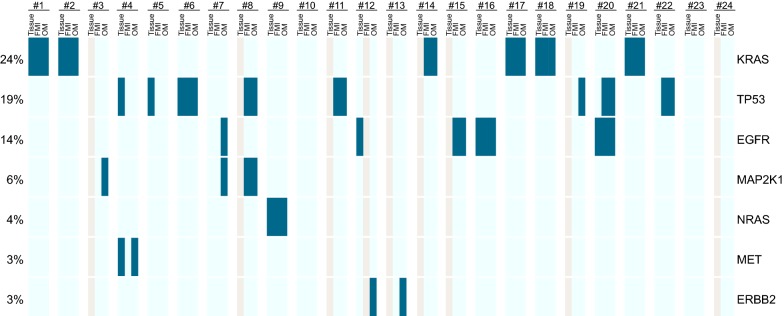
Mutation assessment using the in-house approach (Oncomine cfTNA = OM) and the outsourced test (Foundation Liquid = FMI) as well as the tissue-based NGS (tissue). Results are grouped for each patient (number at top; 1–24) and genes are shown in each row (the frequency of mutations per gene are highlighted at the left side next to each gene). Only genes with detected mutations are shown. The light grey color marks tests which failed or were impossible to perform

As the different panels span different regions of the genes, only overlapping regions were used for the concordance analysis (Additional file 1: Figure S1). For the overlapping regions, 16 mutations were detected in total using the out-sourced test, all of which were confirmed by the in-house test (Fig. 1). However, six additional mutations have been detected using the in-house test which were not confirmed by the outsourced test plus one sample where the outsourced data is missing (Fig. 1). Consequently, the concordance rate between the two panels was 73% (16/22 concordant mutations). Interestingly all the mutations that were additionally called by the in-house test had an allele frequency of < 0.35% except one Her2 exon 20 insertion (AF = 1.89%). However, one *EGFR* mutation (G719A) was not detected by tissue NGS and targeted based PCR (Idylla) and was not confirmed by the outsourced test but was only detected by the in-house test at a very low allele frequency of 0.08% might consequently be a false positive call.

In comparison to the 16 samples where tissue NGS was present, thirteen mutations were detected, of which nine were confirmed using the outsourced test (for one sample no data was present so 9/15 mutations [60%] were confirmed) and ten using the in-house test (10/16; 63%). Interestingly, all the mutations that were only detected in the tissue were the TP53 mutation, which indicates a certain challenge in detecting them in plasma. However, one druggable *EGFR* del19 was only detected in tissue and not in the in-house liquid biopsy assays (Fig. 1).

Importantly, the outsourced test covers more genes and spans more regions than the in-house test, so consequently more mutations have been detected in the outsourced test (Additional file 1: Figure S4).

The correlation of the allele frequency of mutations detected by the two tests was very high (R^2^ = 0.984) and even for mutations with an allele frequency below 2%, the correlation was still very stable (R^2^ = 0.633; Additional file 1: Figure S5).

### Impact of NGS from liquid biopsies on therapeutic strategy

**Fig. 2:**
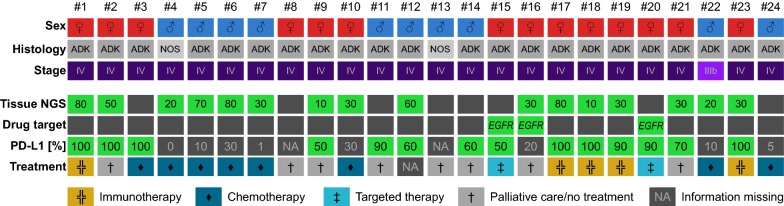
Impact of liquid biopsy NGS testing on treatment selection. Each column represents one patient. Tissue NGS was possible in 16/24 patients and PD-L1 expression was assessed in 22/24 patients. The percentage of tumor cells in the tissue is indicated as a number for each patient in the tissue NGS row. Treatment selection was based on the PD-L1 expression from tissue sections and the detection of targetable EGFR mutations. While the PD-L1 expression in patient #3 would have allowed the initiation of first-line treatment with the anti-PD1 antibody pembrolizumab, there were clinical reasons prohibiting the treatment and consequently the patient was initially treated with chemotherapy. *ADK* adenocarcinoma, *NOS* non-small cell lung cancer not otherwise specified

Patients in this study were included prospectively and the impact of the NGS testing from liquid biopsies on the treatment decision was assessed (Fig. 2). An activating *EGFR* mutation (exon 19 deletion) was detected in three patients and a targeted treatment (EGFR tyrosine kinase inhibitor erlotinib) was initiated in two of them, while the third patient died because of disease progression before the treatment could be initiated (Fig. 2). For the two other patients, no tissue NGS test was possible and the LB test was the only test to detect the *EGFR* mutation in the respective patients. Five of 24 patients (21%) were treated with immunotherapy (anti-PD1 checkpoint inhibitor pembrolizumab) in first line. Treatment decisions were not based on the LB results but on PD-L1 IHC showing more than 50% positive tumor cells in tissue sections (Fig. 2). 8/24 (33%) patients were treated with chemotherapy (platinum-doublet therapy) based on the low PD-L1 expression in the associated tissue sections. Additionally, 8/24 (33%) patients did not start an anti-cancer treatment due to the very advanced stage of their cancer or because they died before possible treatment initiation (Fig. 2).

## Discussion

This study shows that it is feasible to integrate NGS testing from liquid biopsies in molecular tumor assessment made at baseline in late-stage NSCC using an in-house approach and an outsourced test. Importantly, both panels were able to assess genomic mutations and the allele frequency was highly correlated (Fig. 1, Additional file 1: Figure S5; Additional file 2: Table S2). It is noteworthy that the in-house test was based on 2 ml of plasma while the outsourced test required significantly more sample input (17 ml of whole blood). Consequently, the in-house test might be favorable when blood samples from the patients are very limited, especially as the in-house test was able to report mutations down to 0.1% allele frequency highlighting that the limited plasma volume did not affect the sensitivity of the assay. Nevertheless, some mutations were not confirmed across all tests, most importantly, those with a very low allele frequency in plasma. Based on the small sample cohort it very challenging to determine if those mutations that were not confirmed by other tests are false-positive calls or, alternatively, are due to an underlying intratumoral heterogeneity that hindered their detection in tissue NGS. However, our results are in line with a recent study comparing ctDNA and tissue DNA sequencing by Sabari et al. which highlighted a rather limited concordance between plasma and tissue based sequencing [13] but are lower than another study by Guibert et al. [14].

Importantly, one targetable *EGFR* mutation was detected in tissue only, while another *EGFR* mutation was detected at very low frequency using the in-house liquid biopsy assay only. This needs further investigation as the detection of targetable mutations is of utmost importance for patient care.

Furthermore, only the in-house test reported a *MET* I1010T mutation that was not a somatic mutation of the tumor but a germline polymorphism that is known to impact the function of the protein [15]. Consequently, it would have been beneficial if this polymorphism was also reported by the outsourced test. However, the mutation admittedly has no current therapeutic impact.

Importantly, tissue-based NGS or sequential molecular testing from tissue section was not possible in 8/24 (33%) patients due to a low amount of extracted DNA or the absence of tissue left for the analysis. Here, the liquid biopsy sequencing was the only test that was performed to look for druggable mutations. Indeed, in two of the patients where no tissue testing was possible, an *EGFR* exon 19 deletion has been detected and a targeted treatment was initiated, highlighting the usability of this approach. However, *EGFR* mutations can easily be detected using PCR-based assays, so the same results could have been obtained using such an approach [3]. However, with the development of novel targeted treatment options in lung cancer, the NGS approach might become more relevant in order to cover all the requested biomarkers. Additionally, plasma-based NGS is able to not only detect targetable mutations at baseline but also minimal residual disease at progression and it might consequently be beneficial as a prognostic tool [16, 17].

In our study, the turn-around time of the outsourced test was significantly shorter than the in-house testing as patient inclusion especially at the beginning of the study was slow, requiring longer duration to initiate a sequencing run. However, higher patient number will certainly dramatically reduce the turn-around time of the in-house testing. Nevertheless, the outsourced test might be more beneficial when a lower patient number is expected to be tested. However, testing results are often urgent and the in-house test should allow processing the whole sequencing run in 3 days, if needed, which is not possible with the outsourced test.

Importantly, our study is limited in patient number and we consequently cannot draw universal conclusions based on our results. Additionally, the sensitivity of the assay is strongly dependent on the amount of isolated nucleic acids which was very variable between the patients. In this regard it has been shown that the amount of circulating tumor DNA is dependent on tumor burden and disease stage which might explain the reduced sensitivity in some patients [18]. Interestingly, the recommended biomarkers in late-stage NSCC with an approved targeted treatment are *EGFR*, *BRAF*, *ALK*, *ROS1*, and more recently *NTRK* [1]. The latter three are genomic rearrangements that are assessed using extracted RNA rather than extracted DNA. The in-house NGS LB assays used in the study includes DNA and RNA extracted from plasma for enhanced detection of rearrangements, but no patients with relevant alterations in these biomarkers were detected in our study which is not surprising considering their low prevalence [19]. In a recent study conducted at the MD Anderson Cancer Center (Houston, Texas), it was concluded that NGS from LB can completely replace tissue-based testing at a reduced TAT but only a few genomic rearrangements were included in the study population [20]. Recently, the feasibility of the detection of *ALK* rearrangements from plasma samples has been demonstrated using the same outsourced test as reported in the present study which highlights the current advanced in implementing liquid biopsy-based sequencing at baseline [21]. However, the sensitivity of LB for the detection of genomic rearrangements, in comparison to tissue-based testing, needs urgent confirmation.

To initiate a treatment with immune-checkpoint inhibitors (ICIs), like pembrolizumab, the expression of PD-L1 in tumor cells must be assessed and it has to be superior to 50% [22]. Indeed, first line pembrolizumab treatment was initiated in 5/24 (21%) patients in our cohort, based on a tissue biopsy and not the LB results as no PD-L1 assay had been validated in daily practice using blood samples until now. Consequently, the treatment selection in our cohort was mainly driven by PD-L1 IHC on tissue sections and not by the data obtained from LB, thus highlighting the current limitations on the use of plasma-based NGS alone in NSCC patients. However, NGS from plasma certainly has some advantages over current PCR-based methods, as it allows one to assess other biomarkers, like the tumor mutational burden that has been demonstrated to be predictive for response under immunotherapy [23].

## Conclusion

In conclusion, NGS- based LB is a promising new approach for the assessment of genomic biomarkers for the stratification of late-stage NSCC patients at baseline in routine clinical care. The development of novel targeted treatments in this setting will dramatically expand its use in daily practice. However, the importance of PD-L1 IHC as the only approved biomarker for the stratification of patients undergoing ICI treatment until the present day, as well as the currently unknown sensitivity for the testing of genomic rearrangements, makes it a mandatory addition to tissue-based testing from biopsies. In our opinion, plasma-based NGS cannot currently replace tissue testing at baseline in routine clinical and molecular pathology.

## Supplementary information

**Additional file 1: Table S1.** Patient characteristics. **Figure S1.** Genes covered by the respective panels used. The Oncomine cfTNA and the Hotspot V2 panel only covers selected exons in the respective genes. For the Foundation Liquid assay: Ø the whole exonic region of the respective gene is covered. ¥ Only selected regions are covered by the assay. For genes highlighted in blue, genomic rearrangements are also detected. **Figure S2.** Relationship between cfDNA concentration and detection limit. The minimal detection limit (in  % of allele frequency) is highlighted for each sample depending on the measured cfDNA concentration after nucleic acid extraction. Detection limit improves with increased sample input. One sample with a very high cfDNA concentration of 76.6 ng/µl is not shown on the figure for better graphical presentation. **Figure S3.** Density plot of the turn-around time for the outsourced test. The time needed for the sending of the samples to the certified testing center and the time needed at the testing center to sequence the sample and generate the report is highlight as well as the total time needed. Time is shown in business days assuming a Monday–Friday working week with both days inclusive for the calculation. Each bar below the curve highlights one sample. **Figure S4.** Mutations detected using the outsourced test (Foundation Liquid). As the outsourced test covers more genes and spans more regions in the respective some additional mutations have been detected in the patients. Each line represents one gene (the frequency of mutations per gene are highlighted at the left side next to each gene) and each column one patient. The different types of genetic alterations are color coded. **Figure S5.** Correlation of allele frequency between the two liquid biopsy assays. **A** The correlation for all mutations that were found in the two tests is shown. **B** The correlation of the mutations in a subset where the allele frequency assessed by the Foundation Liquid test was < 2%.

**Additional file 2.** Table of mutations detected by the respective panels.

## Data Availability

Sequencing data is available as Additional file 2: Table S2. Other data will be made available upon reasonable request to the corresponding author.

## Abbreviations

NSCC: Non-squamous cell lung carcinoma
LB: Liquid biopsies
TAT: Turn-around time
TNA: Total nucleic acids
FFPE: Formalin-fixed and paraffin-embedded
ICI: Immune-checkpoint inhibitor

## References

[1] Planchard D, Popat S, Kerr K (2018). Metastatic non-small cell lung cancer: ESMO Clinical Practice Guidelines for diagnosis, treatment and follow-up. Ann Oncol..

[2] Rothwell DG, Ayub M, Cook N (2019). Utility of ctDNA to support patient selection for early phase clinical trials: the TARGET study. Nat Med.

[3] Heeke S, Benzaquen J, Hofman V (2019). Critical assessment in routine clinical practice of liquid biopsy for EGFR status testing in non-small-cell lung cancer: a single-laboratory experience (LPCE, Nice, France). Clin Lung Cancer..

[4] Li BT, Janku F, Jung B (2019). Ultra-deep next-generation sequencing of plasma cell-free DNA in patients with advanced lung cancers: results from the Actionable Genome Consortium. Ann Oncol.

[5] Aggarwal C, Thompson JC, Black TA (2019). Clinical implications of plasma-based genotyping with the delivery of personalized therapy in metastatic non-small cell lung cancer. JAMA Oncol..

[6] Teder H, Koel M, Paluoja P (2018). TAC-seq: targeted DNA and RNA sequencing for precise biomarker molecule counting. Npj Genomic Med..

[7] Targeted sequencing for liquid biopsy cancer research. 2019 [cited 2019 Oct 31]. https://assets.thermofisher.com/TFS-Assets/CSD/Flyers/liquid-biopsy-cell-free-research-assays-flyer.pdf.

[8] Cofrac. Accreditation Certificate No 8-3034 rév. 10. 2018. https://tools.cofrac.fr/annexes/sect8/8-3034.pdf.

[9] Ilie M, Butori C, Lassalle S (2017). Optimization of EGFR mutation detection by the fully-automated qPCR-based Idylla system on tumor tissue from patients with non-small cell lung cancer. Oncotarget..

[10] Heeke S, Hofman V, Long-Mira E (2018). Use of the ion PGM and the genereader NGS systems in daily routine practice for advanced lung adenocarcinoma patients: a practical point of view reporting a comparative study and assessment of 90 patients. Cancers (Basel)..

[11] Technical Specifications. 2019 [cited 2019 Oct 31]. https://www.foundationmedicine.nl/content/dam/rfm/nl_v2-en_nl/Documents/NL_Technical_information_Liquid.pdf.

[12] Fehske H., Schneider R., Weiße A. (2008). Computational Many-Particle Physics.

[13] Sabari JK, Offin M, Stephens D (2019). A prospective study of circulating tumor dna to guide matched targeted therapy in lung cancers. JNCI J Natl Cancer Inst..

[14] Guibert N, Hu Y, Feeney N (2018). Amplicon-based next-generation sequencing of plasma cell-free DNA for detection of driver and resistance mutations in advanced non-small cell lung cancer. Ann Oncol.

[15] Liu S, Meric-Bernstam F, Parinyanitikul N (2015). Functional consequence of the MET-T1010I polymorphism in breast cancer. Oncotarget..

[16] Pantel K, Alix-Panabières C (2019). Liquid biopsy and minimal residual disease—latest advances and implications for cure. Nat Rev Clin Oncol..

[17] Chaudhuri AA, Chabon JJ, Lovejoy AF (2017). Early detection of molecular residual disease in localized lung cancer by circulating tumor DNA profiling. Cancer Discov.

[18] Newman AM, Bratman SV, To J (2014). An ultrasensitive method for quantitating circulating tumor DNA with broad patient coverage. Nat Med.

[19] Skoulidis F, Heymach JV (2019). Co-occurring genomic alterations in non-small-cell lung cancer biology and therapy. Nat Rev Cancer..

[20] Leighl NB, Page RD, Raymond VM (2019). Clinical utility of comprehensive cell-free dna analysis to identify genomic biomarkers in patients with newly diagnosed metastatic non–small cell lung cancer. Clin Cancer Res.

[21] Gadgeel SM, Mok TSK, Peters S (2019). LBA81_PRPhase II/III blood first assay screening trial (BFAST) in patients (pts) with treatment-naïve NSCLC: Initial results from the ALK + cohort. Ann Oncol..

[22] Reck M, Rodríguez-Abreu D, Robinson AG (2016). Pembrolizumab versus chemotherapy for PD-L1–positive non–small-cell lung cancer. N Engl J Med.

[23] Gandara DR, Paul SM, Kowanetz M (2018). Blood-based tumor mutational burden as a predictor of clinical benefit in non-small-cell lung cancer patients treated with atezolizumab. Nat Med.

