# Supplementary material for: Prospective evaluation of NGS-based liquid biopsy in untreated late stage non-squamous lung carcinoma in a single institution
Source: J Transl Med. 2020 Feb 17;18:87. doi: 10.1186/s12967-020-02259-2 (PMC7027049; doi:10.1186/s12967-020-02259-2)
Supplement: Supplementary file 1 — Additional file 1: Table S1. Patient characteristics. Figure S1. Genes covered by the respective panels used. The Oncomine cfTNA and the Hotspot V2 panel only covers selected exons in the respective genes. For the Foundation Liquid assay: Ø the whole exonic region of the respective gene is covered. ¥ Only selected regions are covered by the assay. For genes highlighted in blue, genomic rearrangements are also detected. Figure S2. Relationship between cfDNA concentration and detection limit. The minimal detection limit (in % of allele frequency) is highlighted for each sample depending on the measured cfDNA concentration after nucleic acid extraction. Detection limit improves with increased sample input. One sample with a very high cfDNA concentration of 76.6 ng/µl is not shown on the figure for better graphical presentation. Figure S3. Density plot of the turn-around time for the outsourced test. The time needed for the sending of the samples to the certified testing center and the time needed at the testing center to sequence the sample and generate the report is highlight as well as the total time needed. Time is shown in business days assuming a Monday–Friday working week with both days inclusive for the calculation. Each bar below the curve highlights one sample. Figure S4. Mutations detected using the outsourced test (Foundation Liquid). As the outsourced test covers more genes and spans more regions in the respective some additional mutations have been detected in the patients. Each line represents one gene (the frequency of mutations per gene are highlighted at the left side next to each gene) and each column one patient. The different types of genetic alterations are color coded. Figure S5. Correlation of allele frequency between the two liquid biopsy assays. A The correlation for all mutations that were found in the two tests is shown. B The correlation of the mutations in a subset where the allele frequency assessed by the Foundation Liquid test was < 2%. [file 12967_2020_2259_MOESM1_ESM.docx]

**Additional file 1**

**Table S1**: Patient characteristics.

|  | **Patients** |
| --- | --- |
| **N** | 24 |
| **Age** |  |
| Median (Range) | 63 years (39 – 85) |
| **Sex** |  |
| Female | 14 |
| Male | 10 |
| **Smoking** |  |
| Active | 15 |
| Former | 7 |
| Never | 2 |
| **Histological subtype** |  |
| Adenocarcinoma | 23 |
| NSCLC^1^ not otherwise specified | 1 |
| **Stage** |  |
| IIIb | 1 |
| IV | 23 |
| **ECOG^2^ Performance status** |  |
| 0-2 | 16 |
| 3-4 | 6 |
| Unknown | 2 |
| **Brain metastasis at diagnosis** |  |
| Present | 6 |
| Absent | 18 |

1 NSCLC = Non-small cell lung cancer; 2 ECOG = Eastern Cooperative Oncology Group


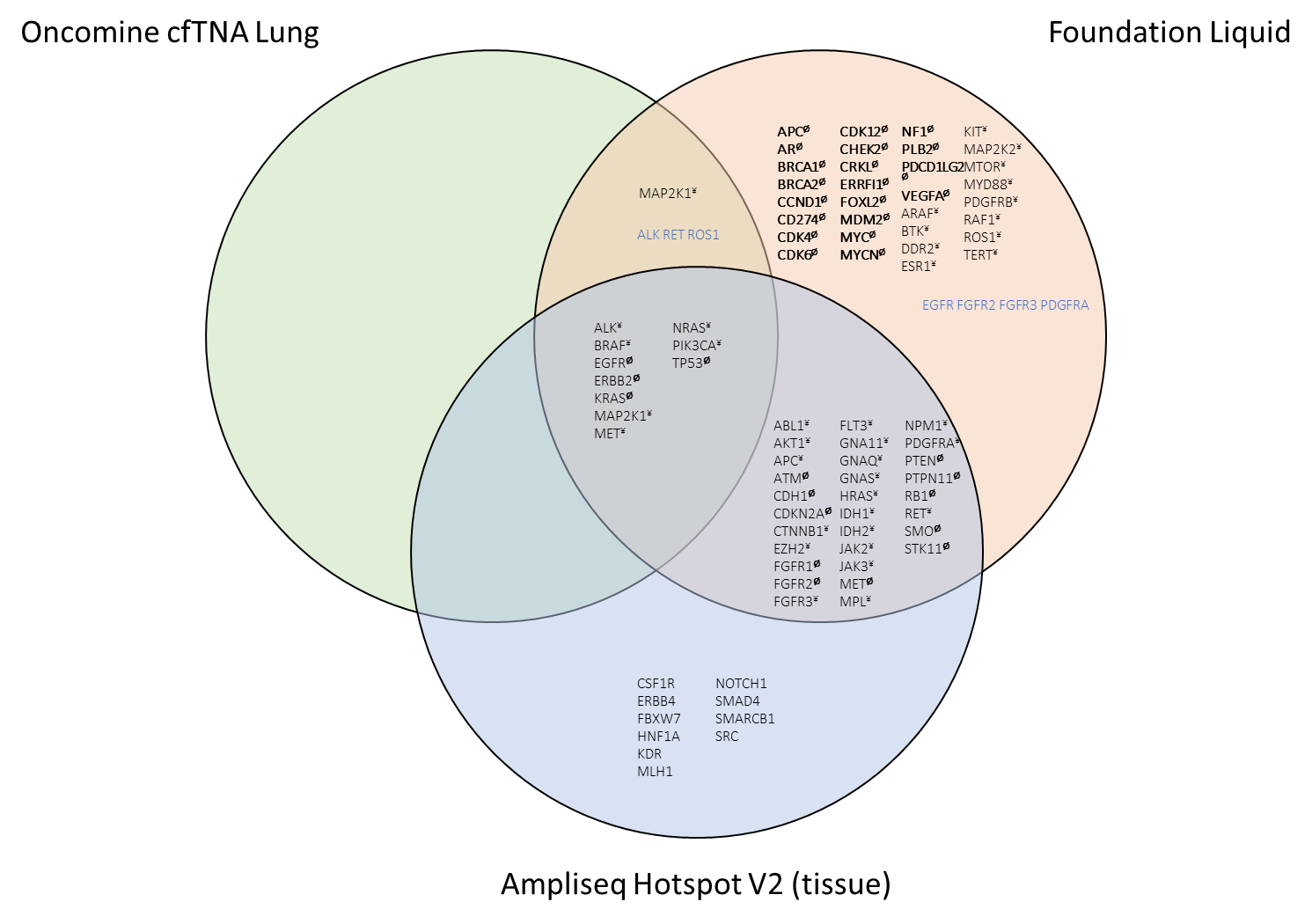


**Figure S1**: Genes covered by the respective panels used. The Oncomine cfTNA and the Hotspot V2 panel only covers selected exons in the respective genes. For the Foundation Liquid assay: Ø the whole exonic region of the respective gene is covered. ¥ Only selected regions are covered by the assay. For genes highlighted in blue, genomic rearrangements are also detected.


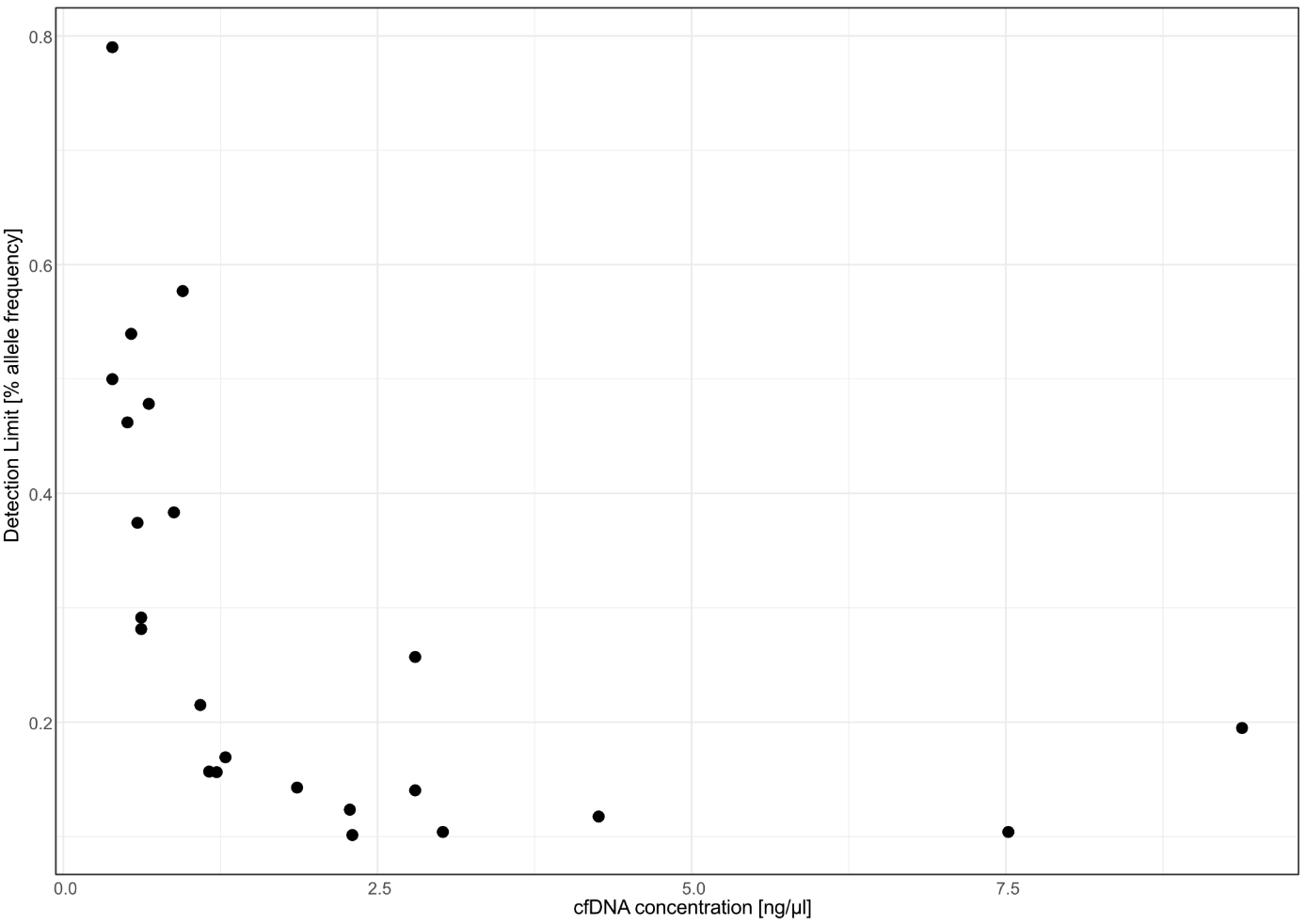


**Figure S2:** Relationship between cfDNA concentration and detection limit. The minimal detection limit (in % of allele frequency) is highlighted for each sample depending on the measured cfDNA concentration after nucleic acid extraction. Detection limit improves with increased sample input. One sample with a very high cfDNA concentration of 76.6 ng/µl is not shown on the figure for better graphical presentation.


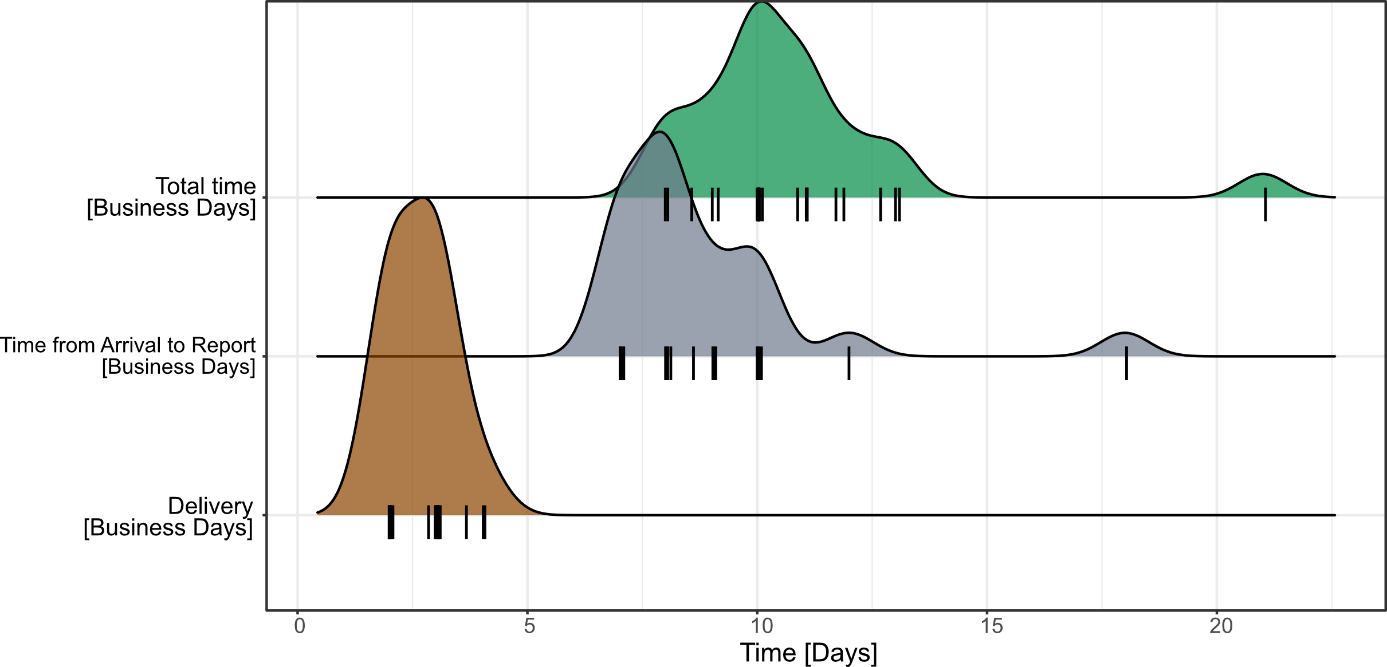


**Figure S3**: Density plot of the turn-around time for the outsourced test. The time needed for the sending of the samples to the certified testing center and the time needed at the testing center to sequence the sample and generate the report is highlight as well as the total time needed. Time is shown in business days assuming a Monday - Friday working week with both days inclusive for the calculation. Each bar below the curve highlights one sample.


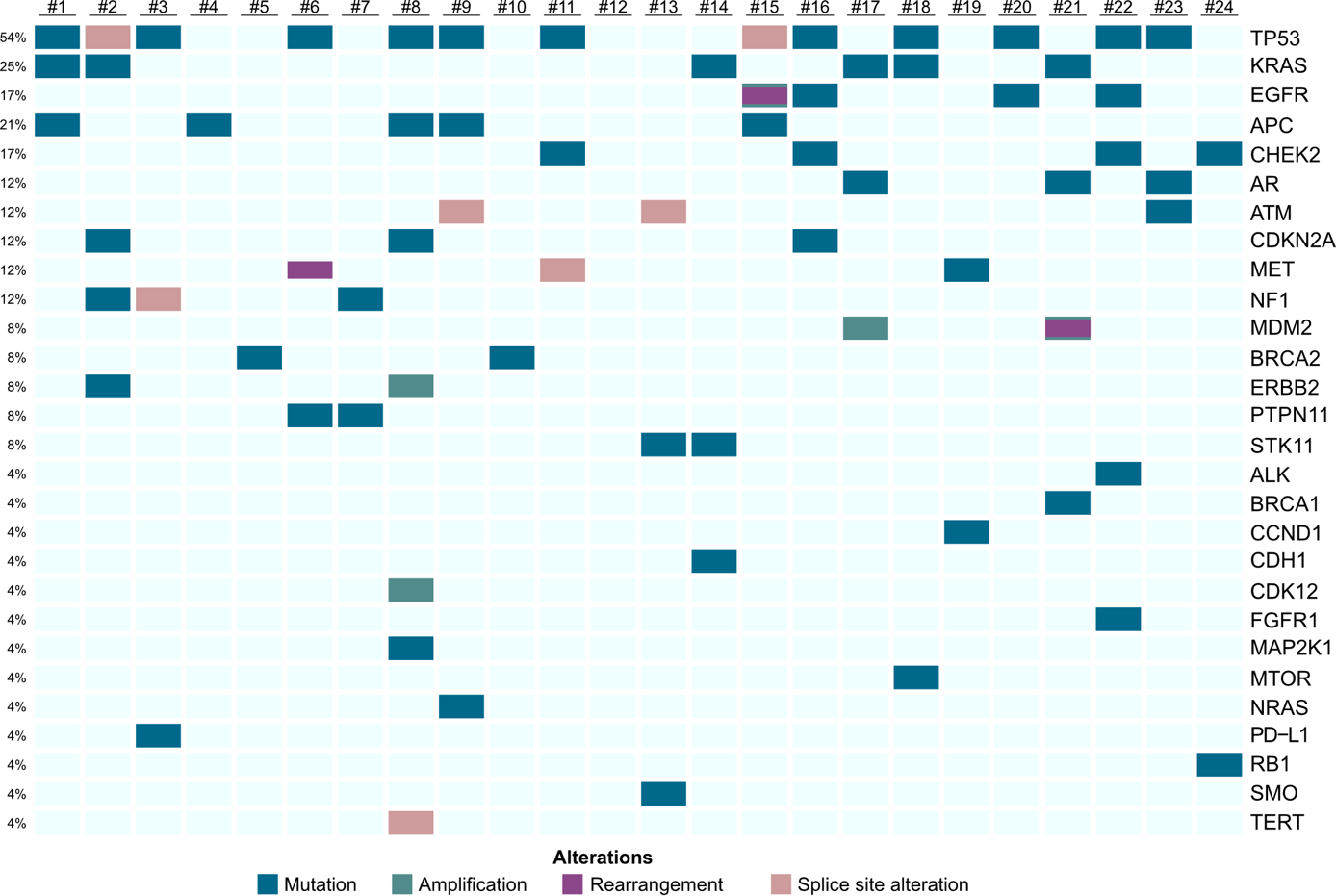


**Figure S4:** Mutations detected using the outsourced test (Foundation Liquid). As the outsourced test covers more genes and spans more regions in the respective some additional mutations have been detected in the patients. Each line represents one gene (the frequency of mutations per gene are highlighted at the left side next to each gene) and each column one patient. The different types of genetic alterations are color coded.


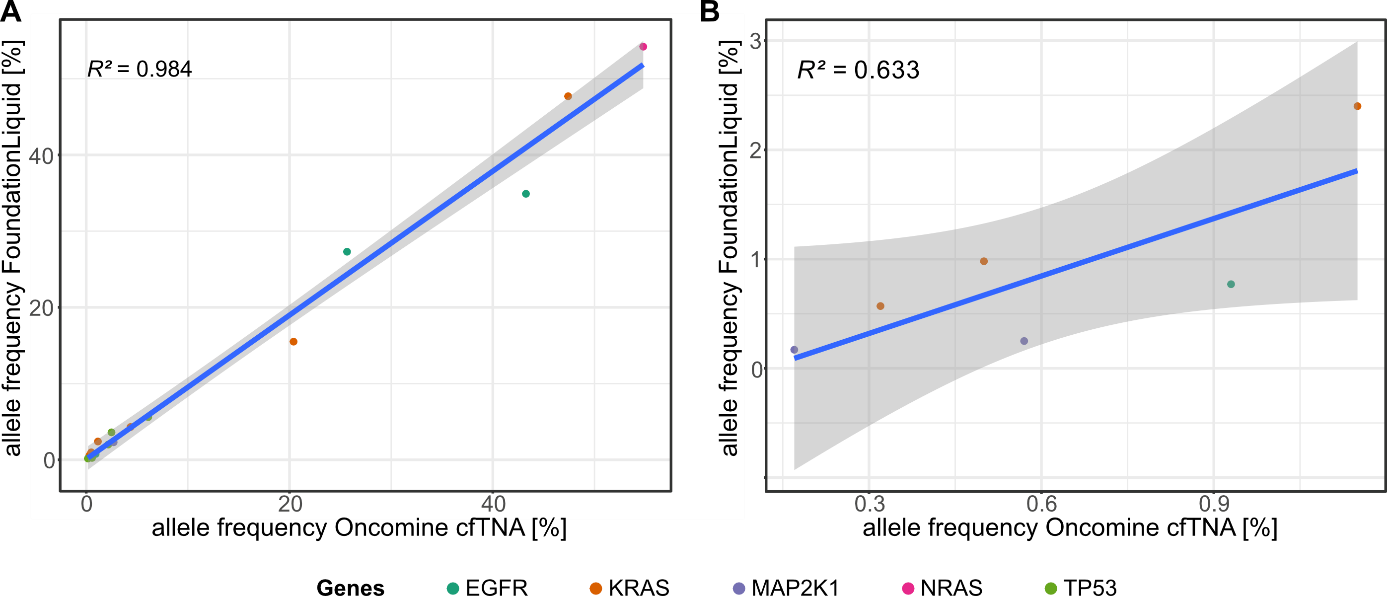


**Figure S5**: Correlation of allele frequency between the two liquid biopsy assays. **A** The correlation for all mutations that were found in the two tests is shown. **B** The correlation of the mutations in a subset where the allele frequency assessed by the Foundation Liquid test was < 2%.
